# Antiviral Activity of *Fritillaria thunbergii* Extract against Human Influenza Virus H1N1 (PR8) In Vitro, *In Ovo* and In Vivo

**DOI:** 10.4014/jmb.1908.08001

**Published:** 2019-11-18

**Authors:** Minjee Kim, Dinh-Van Nguyen, Yoonki Heo, Ki Hoon Park, Hyun-Dong Paik, Young Bong Kim

**Affiliations:** 1 Department of Biomedical Science and Engineering, Konkuk University, Seoul 05029, Republic of Korea; 2 Department of Food Science and Biotechnology of Animal Resources, Konkuk University, Seoul 0509, Republic of Korea

**Keywords:** Antiviral activity, influenza A (H1N1), *Fritillaria thunbergii*, oseltamivir

## Abstract

Influenza viruses cause respiratory diseases in humans and animals with high morbidity and mortality rates. Conventional anti-influenza drugs are reported to exert side effects and newly emerging viral strains tend to develop resistance to these commonly used agents. *Fritillaria thunbergii* (FT) is traditionally used as an expectorant for controlling airway inflammatory disorders. Here, we evaluated the therapeutic effects of FT extracts against influenza virus type A (H1N1) infection in vitro, *in ovo*, and in vivo. In the post-treatment assay, FT extracts showed high CC_50_ (7,500 μg/ml), indicating low toxicity, and exerted moderate antiviral effects compared to oseltamivir (SI 50.6 vs. 222) in vitro. Antiviral activity tests *in ovo* revealed strong inhibitory effects of both FT extract and oseltamivir against H1N1 replication in embryonated eggs. Notably, at a treatment concentration of 150 mg/kg, only half the group administered oseltamivir survived whereas the FT group showed 100% survival, clearly demonstrating the low toxicity of FT extracts. Consistent with these findings, FT-administered mice showed a higher survival rate with lower body weight reduction relative to the oseltamivir group upon treatment 24 h after viral infection. Our collective results suggest that FT extracts exert antiviral effects against influenza H1N1 virus without inducing toxicity in vitro, *in ovo* or in vivo, thereby supporting the potential utility of FT extract as a novel candidate therapeutic drug or supplement against influenza.

## Introduction

The significant global impact of influenza infection is driven by the evolution of diverse viral strains, with outbreaks occurring frequently among high-risk populations. The identification of human influenza A (H1N1) has raised awareness of pandemic threats and led to the extensive development of vaccines and antiviral agents for its control [1]. Since the outbreak of “Spanish flu” in 1918 that resulted in about 50 million deaths, the long-term epidemic of H1N1 has been attributed to its rapid genetic mutation to escape host immune protection [2]. In view of the emergence of drug-resistant viruses associated with clinical manifestations, such as fever and upper respiratory symptoms, a rational approach is critical for management of the next possible pandemic event [3].

All influenza viruses have two characteristic surface glycoproteins, specifically, hemagglutinin and neuraminidase [4]. Hemagglutinin is a sialic acid receptor that mediates virus entry and neuraminidase cleaves the newly formed virus from infected host cells [1]. Neuraminidase may also facilitate viral invasion of the upper airways, possibly by cleaving the sialic acid moieties on mucin secreted by airway epithelial cells [5]. To date, only two classes of anti-influenza drugs have been approved: (1) M2 ion channel inhibitors (amantadine and rimantadine) and (2) neuraminidase inhibitors (oseltamivir or zanamivir). During viral entry, the M2 protein promotes uncoating of the influenza virus after membrane fusion and mediates acidification of the intracellular compartments of viral particles in endosomes [6, 7]. Therefore, inhibition of the M2 ion channel interferes with viral uncoating and fusion [6]. However, the emergence of resistant variants and toxic effects limit the clinical use of amantadine and rimantadine [8]. The neuraminidase inhibitor, oseltamivir, is most commonly employed to treat influenza virus infection. Neuraminidase inhibitors interfere with virus release from host cells, preventing infection of new host cells [1]. Despite good tolerance of this drug by most patient groups, there have been reports of neuropsychiatric side effects, especially in young patients [9], although the contributory factors are presently unknown [10].

Due to the toxic side effects of conventional antiviral drugs and antigenic drift in viruses, the development of novel effective treatment options for influenza remains an urgent health challenge worldwide. Research attention has recently focused on natural products as potential sources of widely accessible antiviral drug candidates with low toxicity. The bulbs of *Fritillaria thunbergii* (FT) belonging to the lily family have been traditionally used as expectorants for controlling airway inflammatory diseases and treatment of coughs, bronchitis, pneumonia, and fever-based illnesses [11, 12]. In this study, we examined the hypothesis that FT should not only reduce the respiratory symptoms associated with influenza but also inhibit influenza virus activity. We further compared the therapeutic effects of FT against influenza A to those of the conventional anti-influenza drug oseltamivir in vitro, *in ovo*, and in vivo.

## Materials and Methods

### Cells, Viruses and Preparation of FT Extracts

Mardin-Darby Canine Kidney (MDCK) cells were obtained from the Korean Cell Line Bank (Korea) and maintained in MEM supplemented with 10% fetal bovine serum (FBS), 100 U/ml penicillin and 100 µg/ml streptomycin at 37°C in 5% CO_2_. Human influenza virus type A/PR/8/1934 (H1N1) was provided by the Centers for Disease Control and Prevention (CDC, Korea). Viruses were amplified and maintained via passage in 9-day-old embryonated chicken eggs. After three days of incubation at 37°C and chilling at 4°C for 12 h, allantoic fluid was harvested and maintained at -80°C until use.

Dried herbs were ground and extracted with boiling water (10 volumes/g) for 3 h. Liquid extracts were filtered while powder extracts were dissolved in serum-free Eagle’s minimum essential medium (MEM-Gibco) to obtain a concentration of 100 mg/ml. The solution was sterilized via filtration using a 0.45 µm pore size membrane filter for assay.

### Cytotoxicity Assay

Cell viability was examined with the methylthiazol tetrazolium (MTT) assay. MDCK cells were seeded at a density of 1.5 × 10^4^ cells/ml in a 96-well plate and incubated at 37°C for 24 h. Cell monolayers were washed twice with 100 µl PBS, exposed to 100 µl medium containing serially diluted extracts of each sample and incubated in 5% CO_2_ at 37°C for 48 h. Aliquots of 20 µl/well MTT (VWR, USA) were added to each plate followed by incubation in the dark for 2 h, with assessment of purple formazan crystal formation every 30 min. Once proper formazan crystal formation was observed, contents of the wells were completely aspirated.

Immediately after aspiration, 100 µl/well of 100% DMSO was added to each plate and mixed at room temperature for 30 min. Absorbance at 570 nm was read on a microplate reader (Beckman Coulter ADSA). The cytotoxicity concentration at 50% value (CC_50_) of each compound was calculated via regression analysis.

### Cytopathic Effect (CPE) Reduction Assay

Antiviral effects in vitro were evaluated using the cytopathic effect reduction assay. Briefly, a confluent monolayer of MDCK cells was washed twice with PBS and infected with 100 TCID_50_ virus for 2 h. Following the incubation period, unabsorbed virus was removed by washing. Cell plates were incubated with virus growth medium (MEM) containing 0.3% bovine serum albumin, 1% Penicillin–Streptomycin solution and trypsin treated with L-1-tosylamido-2-phenylethyl chloromethyl ketone (trypsin-TPCK; 1 µg/ml) containing serially diluted herbal extracts for 48 h at 37°C until evidence of viral CPE. In each sheet, controls infected with 100 TCID_50_ virus, in addition to uninfected and untreated mock controls, were included for all experiments.

The antiviral activity of each compound was estimated based on viral CPE reduction ability and expressed as half maximal viral inhibition effective concentration (EC_50_). Anti-influenza activity was finally expressed as selectivity index (SI), calculated as CC_50_ divided by EC_50_.

### Virus TCID_50_ Determination and Anti-Influenza Activity of *Fritillaria thunbergii* in MDCK Cells (Post-Treatment Assay)

Virus titration was performed using the TCID_50_ assay in MDCK cells according to WHO surveillance guidelines. TCID_50_ determination was performed with the limiting dilution method performed in 96-well microtiter plates with a series of 10-fold dilutions. The virus titer was estimated from the cytopathic effect (CPE) of cells induced by infection and expressed as 50% tissue culture infectious dose (TCID_50_) described by Reed and Muench [13].

Activity of *F. thunbergii* against AIV H1N1 infection was determined using the post-treatment assay. Briefly, MDCK cells were infected with 100 TCID_50_ H1N1 for 2 h at 37°C. After viral inoculation, cells were treated with various concentrations of FT extract for 48 h at 37°C and anti-influenza activity expressed as selectivity index (SI) value.

### *In Ovo* Anti-Influenza Activity of *Fritillaria thunbergii*

Different H1N1 dilutions in 0.2 ml PBS were added to five 9-day-old embryonated hen eggs. Following incubation for 72 h at 37°C, eggs were candled to assess the survival ratio of chick embryos. All eggs were chilled for 12 h at 4°C, allantoic fluid collected and 50% egg infectivity dose (EID50) calculated using the Reed–Muench method.

To examine antiviral activity *in ovo*, 0.1 ml viral suspension in PBS corresponding to 50 EID50 was injected into the allantoic cavity. Infected embryos subsequently received a single dose of FT extract in PBS (0.1 ml) at different concentrations. Oseltamivir and PBS were used as the positive and negative control, respectively. After incubation at 37°C for 72 h, all eggs were candled to calculate the survival ratio of chick embryos. After chilling at 4°C for 12 h, allantoic fluid in eggs was harvested for virus titration.

### Hemagglutination Assay (HA)

Virus titration was performed with the hemagglutination assay (HA) according to WHO surveillance guidelines (1998). Serial two-fold dilutions of specimens were generated in 50 µl PBS on 96-well U-bottom plates and 50 µl of 0.5% (v/v) chicken erythrocytes in PBS added to each well. After 1 h incubation, HA titers were determined. One HA unit (HAU) was defined as the quantity of virus contained in 1 ml virus suspension of HA titer 1.

### In Vivo Antiviral Assay 

This study was performed in strict accordance with the Guide for the Care and Use of Laboratory Animals of the National Institutes of Health. Animal husbandry and experimental procedures were approved by the Konkuk University Institutional Animal Care and Use Committee (IACUC approval No.: KU16113-1).

Female Balb/C mice aged 8 weeks were purchased from Orient-Bio (Korea). Mice were maintained in a Bio-safety Level 2 facility and housed in cages with water and food under a 12 h day-night cycle. Mice were challenged intranasally and infected with 20MLD_50_ of H1N1 virus strain. Animals were divided (n =10) into placebo, FT-treated (6 mg/mouse), and oseltamivir-treated groups (200 µg/mouse). FT extract and oseltamivir were administered orally via gavage at 4 and 24 h after viral infection followed by twice daily from days 1 to 10 after infection. Survival rates and body weights were monitored for more than two weeks.

### Statistical Analysis 

Statistical analyses were performed using SigmaPlot 11.0 software (Systat Software, USA). Where indicated, statistical significance was assessed using Student’s t-test. *P* values ≤ 0.05 were considered significant. Chi-square test was used to determine a statistical significance between the two categorical variables.

## Results

### Antiviral Activity of FT Extracts against H1N1/PR8 in MDCK Cells (Post-Treatment Assay)

**Fig. 1:**
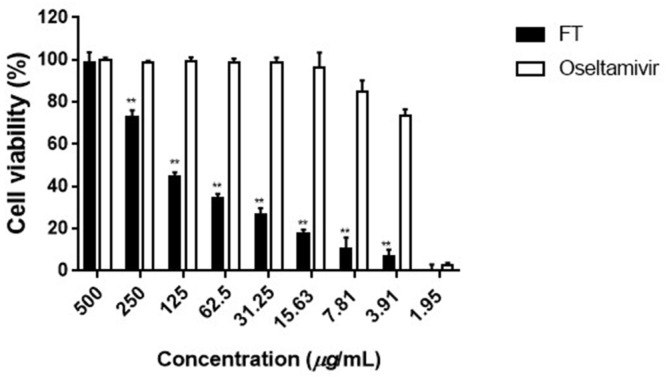
Antiviral effects of FT extracts against H1N1 infection in the post-treatment assay (in vitro). MDCK cells were infected with 100 TCID_50_ H1N1 for 2 h at 37°C and incubated with medium containing different concentrations of FT extracts for 48 h at 37°C. Data are presented as means ± SD from three independent experiments (**p < 0.001 for comparison with oseltamivir groups).

The cytopathic effect (CPE) reduction assay was performed and data expressed as SI values. MDCK cells were infected with 100TCID_50_ H1N1 virus per reaction followed by treatment with different concentrations (1.95–500 µg/ml) of FT extract or oseltamivir for 48 h (post-treatment) (Fig. 1). In the FT extract-treated group, we observed moderate inhibition of H1N1 infection (SI value of 50.6), compared to the oseltamivir treatment group (SI value of 222), indicating a protective effect against influenza replication (Table 1). The CC_50_ of the FT-treated group was ten times higher than that of oseltamivir, demonstrating the low toxicity of FT extracts.

### Anti-Influenza Virus Activity of FT Extracts *In Ovo*

**Fig. 2:**
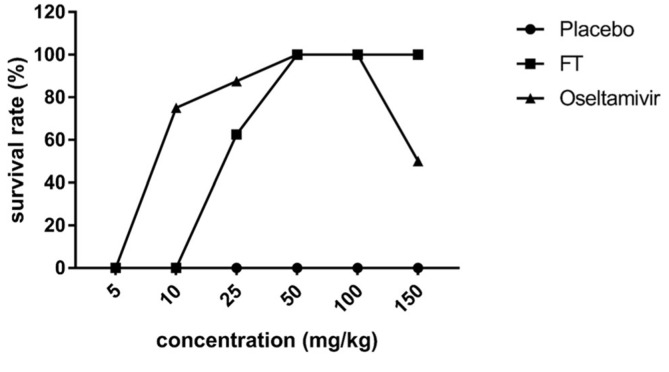
Survival rates of chick embryos after H1N1 infection and treatment with FT extract and oseltamivir (*in ovo*). The viral suspension (0.1 ml of 50EID_50_^-^ was injected into allantoic fluid combined with 0.1 ml *F. thunbergii* extract in PBS) and fluid collected to calculate the survival ratios of chick embryos after incubation at 37°C for 72 h, compared with oseltamivir treatment. Each data point represents the arithmetic mean from 8 eggs per group.

To determine anti-influenza activity *in ovo*, FT extract was mixed into allantoic fluid at concentrations of 10–25 mg/kg in combination with 50EID_50_ H1N1 virus (Fig. 2). The effective dose of oseltamivir was 5 mg/kg whereas that of FT extract was 10 mg/kg. Infected chick embryos showed 100% survival in the presence of both 50 mg/kg FT and oseltamivir. However, at a higher dose of 150 mg/kg, the survival ratio in the oseltamivir treatment group decreased to 50%, suggesting drug toxicity. However, we observed no variations in the survival ratios of embryos subjected to FT extract treatments over 50–150 mg/kg, indicating no toxic effect at these doses of extract.

**Fig. 3:**
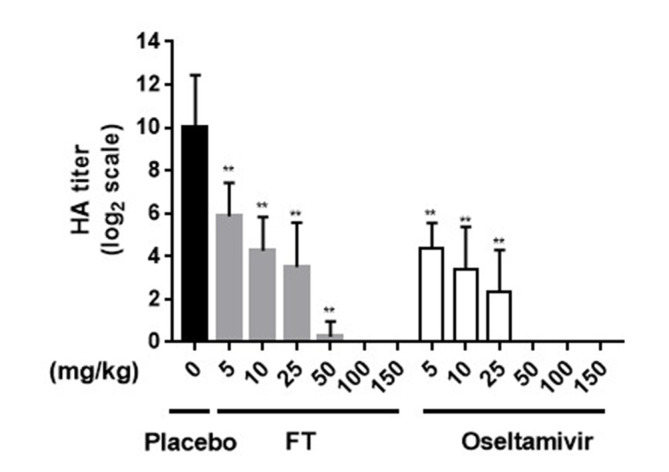
HA titers of chick embryos after H1N1 infection and treatment with FT extract and oseltamivir (*in ovo*). In the FT treatment group, the viral suspension (0.1 ml of 50EID_50_) was injected into allantoic fluid in combination with 0.1 ml FT extract in PBS, and HA titer values compared with those obtained in the oseltamivir and placebo (PBS only) groups (**p < 0.001 for comparison with placebo groups).

To evaluate whether the FT extract affects viremia, the HA titer was measured for each allantoic fluid sample (Fig. 3). Both FT extract and oseltamivir induced a gradual decrease in the HA titer in a concentration-dependent manner. Within a concentration range of 50–150 mg/kg, FT and oseltamivir exerted strong inhibitory effects on H1N1 replication in embryonated eggs.

### Anti-Influenza Virus Activity of FT Extracts In Vivo

**Fig. 4:**
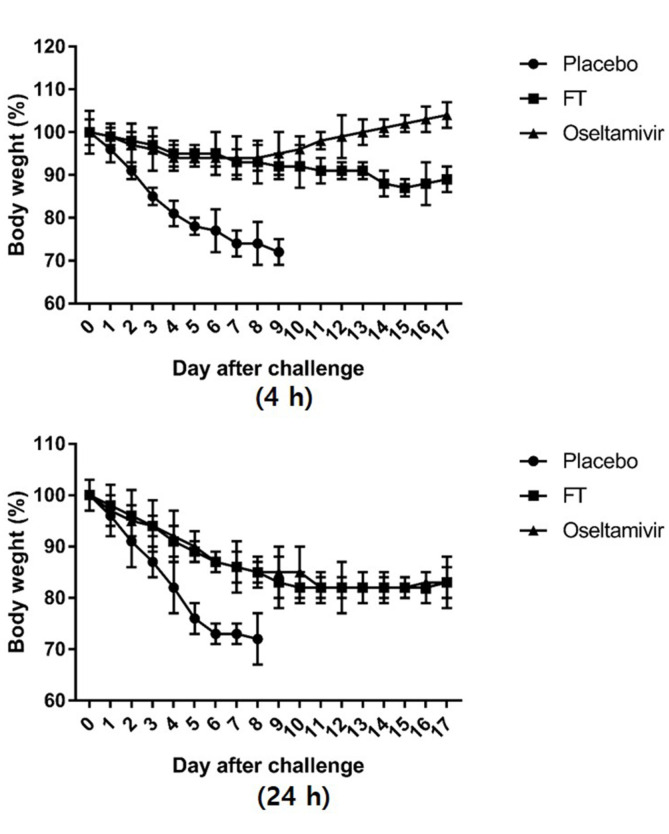
Body weights of mice infected with H1N1 virus and treated with FT extract and oseltamivir at different timepoints (4 h and 24 h). (**A**) Mice (*n* = 10 per group) were intranasally infected with 20MLD_50_ of H1N1 virus strain. FT extracts (6 mg/mouse) or oseltamivir (200 μg/mouse) were administered orally to mice by gavage 4 h after virus infection and treatment continued twice daily from days 1 to 10 post-infection. (**B**) Infected mice were treated with FT extracts or oseltamivir 24 h after virus infection and administration continued twice daily from days 1 to 10 post-infection.

Mice were intranasally infected with 20MLD_50_ of H1N1 virus strain. FT extracts and oseltamivir were administered orally to mice by gavage at 4 h and 24 h after virus infection and treatment continued twice daily from day 1 to 10 (Figs. 4A and 4B). The body weights of mice from the FT-treated group were reduced to 90% whereas those treated with oseltamivir increased in weight by 5%, indicating a superior effect of oseltamivir upon treatment at 4 h after infection (Fig. 4A). Consistent with this finding, 100%survival was observed in the oseltamivir-treated group relative to 40% survival in the FT-treated group (Table 2). However, upon treatment at 24 h after infection, the body weights of FT-treated mice exceeded those on day 5 and surviving mice in the FT group outnumbered those in the oseltamivir group by 3/10 and 1/10, respectively (Fig. 4B, Table 2). All mice in the group treated 36 h after infection died, indicating that treatment within less than 24 h of infection is necessary for survival (data not shown). The placebo group administered PBS showed a continuous reduction in body weight after challenge and all mice died with >30% weight loss by day 6.

## Discussion

Influenza is an acute respiratory disease that causes high morbidity and mortality worldwide [14]. Seasonal influenza infection is a major health burden each year, and despite the successful development of effective antiviral agents, such as amantadine and oseltamivir, a growing risk of resistant virus emergence and side effects limit the use of these drugs to a significant extent. A recent case report in South Korea documented a 22-year-old male complaining of mood swings, suicidal feelings, auditory hallucinations and insomnia after treatment with oseltamivir [9]. A similar case was reported in Japan of a 15-year-old female with influenza who developed abnormal psychiatric symptoms, such as insomnia and visual hallucinations, after administration of standard doses of oseltamivir. Her symptoms disappeared after cessation of oseltamivir treatment and administration of benzodiazepine [10], highlighting the urgent necessity to develop a safe and broadly effective anti-influenza drug.

Plants have a long evolutionary history with respect to developing resistance against viruses and are increasingly investigated as potential sources for development of antiviral drugs [10]. The herb FT is widely distributed throughout northeastern Asia where it is used as traditional medicine to treat respiratory disorder symptoms, such as throat and lung diseases [11]. The bulb has been used as a mucoregulator and expectorant for controlling respiratory disease for decades, and among the various compounds isolated from FT, verticine is reported to exert antitussive and anti-inflammatory effects [11, 12]. The main symptoms of influenza infection include fever, cough and other respiratory illnesses. In view of earlier findings, we hypothesized that FT could effectively relieve influenza-associated respiratory symptoms as well as reduce viral infection.

Data from antiviral assays on MDCK cells suggest that FT extracts exert therapeutic effects against virus by inhibiting the virus replication cycle. Post-treatment assay results showed significant inhibition of H1N1 virus by FT extracts relative to the oseltamivir treatment group (Fig. 1, Table 1), supporting the potential of FT as a therapeutic agent with low toxicity.

Since oseltamivir, the most widely used drug for influenza, acts at the final stage of virus replication, *in ovo* and in vivo studies were further conducted using the post-treatment protocol. *In ovo* results showed that the FT extract exerts an antiviral effect against H1N1 in embryonated eggs without inducing cytotoxicity at a high concentration of 150 mg/kg. At the same dose, oseltamivir exerted toxicity, leading to a 50% decrease in the survival ratio of chick embryos (Fig. 2), suggesting lower toxicity and greater safety of the FT extract, compared to oseltamivir.

In vivo, oseltamivir treatment was associated with 100%survival rate in conjunction with body weight increase whereas the FT extract induced a reduction in both survival and body weight following treatment at 4 h after viral challenge (Fig. 4A, Table 2). However, FT administered 24 h after viral challenge induced a higher survival rate with lower body weight reduction, compared to oseltamivir, supporting its efficacy against viral infection (Fig. 4B, Table 2). Since replication of influenza virus in the respiratory tract reaches peak levels between 24 and 72 h after the onset of illness, drugs such as oseltamivir that act at the viral replication stage must be administered as early as possible [1]. Although the antiviral effects of FT were less pronounced than those of oseltamivir when administered 4 h after viral challenge, a significant finding of this study was that treatment with the extract 24 h after viral challenge led to a higher survival rate and lower body weight loss with lower toxicity than oseltamivir. Therefore, this study discovered that FT has low toxicity and antiviral activity that can be used as a potential candidate for boosting the immune system, or as a supplement for antiviral drugs.

In summary, the aqueous extract of FT exerted therapeutic effects against influenza virus infection in vitro, *in ovo* and in vivo. Importantly, the antiviral effects of the FT extract were induced with no accompanying toxicity in all three experimental systems, compared to the oseltamivir treatment group. To our knowledge, this is the first study to report the antiviral effects of FT against H1N1. FT extract may therefore serve as a potential alternative candidate agent or supplement for treatment of influenza infection to conventional drugs. However, further research is essential to identify the active components and the underlying mechanism against influenza virus and other RNA viruses.

## Figures and Tables

**Table 1:** Anti-viral activity of the herbal extracts against H1N1 in MDCK cells.

Treatment of cells	Compound	CC_50_^a^	EC_50_^b^	SI^c^
Post-treatment assay	FT	7500	148.2	50.6
	Oseltamivir	733	3.3	222

The anti-influenza virus ability of each extract was performed by Cytopathic effect (CPE) reduction assay as described in this report. There is a statistically significant relationship between the categorical variables (Chi-square test *p* < 0.05).

^a^50% cytotoxic concentration (μg/ml)

^b^50% effective concentration (μg/ml)

^c^SI: Selective index, CC_50_/EC_50_

**Table 2:** Survival rates of FT extract- and oseltamivir-treated mice after virus infection at different treatment times.

	4 h		24 h	

	No. of survive/total	Survival rate	No. of survive/total	Survival rate
Placebo	0/10	0%	0/10	0%
FT	4/10	40%	3/10	30%
Oseltamivir	10/10	100%	1/10	10%
